# Clinical performance of different types of dental prosthesis in patients with head and neck tumors—a retrospective cohort study

**DOI:** 10.1007/s00784-022-04673-w

**Published:** 2022-08-17

**Authors:** Karina Zierden, Juliane Wöstmann, Bernd Wöstmann, Peter Rehmann

**Affiliations:** 1 Department of Prosthodontics, School of Dental Medicine, Justus-Liebig-University, Schlangenzahl 14, 35392 Giessen, Germany; 2 Private Practice, Düsseldorf, Nordrhein-Westfalen Germany

**Keywords:** Dental prosthesis, Head and neck tumor, Dental implant, Aftercare, Survival

## Abstract

**Objectives:**

To investigate how different types of dental prosthesis perform in patients with head and neck tumors.

**Materials and methods:**

In this retrospective clinical cohort study, the impact of different patient-related factors was analyzed as influencing factors on the survival probability of dental prosthesis using Kaplan–Meier estimate. For analysis, the dental prosthesis was divided into groups: group 1 (fixed dental prosthesis), group 2 (removable dental prosthesis), group 3 (implant-supported dental prosthesis), and group 4 (prostheses anchored using wrought wire clasps and obturators). The incidental aftercare measures were also evaluated.

**Results:**

Two hundred seventy-nine restorations were observed (mean observation: 2.7 ± 3.0 years, max.14.8 years) out of which 49 (17.6%) had to be replaced during the observation. After 5 years, 100% of group 1 restorations, 79.9% of group 2 restorations, 91.4% of group 3 restorations, and 30% of group 4 restorations were still functional. Four hundred eighty-eight dental implants were observed, of which 77 (15.8%) failed.

**Conclusions:**

Groups 1, 2, and 3 restorations showed good survival times after 5 years in function, whereas group 4 presented the worst survival times. Group 2 restorations showed the highest amount of necessary aftercare measures.

**Clinical relevance:**

The current investigation shows that groups 1, 2, and 3 restorations should be preferred in the prosthetic treatment planning of patients with head and neck tumors. A treatment with group 4 restorations should only be considered if no other prosthetic treatment is possible or as temporary treatment.

## Introduction

The term head and neck tumor includes all tumors located in the lip, oral cavity, nasopharynx, oropharynx, hypopharynx, and larynx area. In 2020, head and neck cancer had again an increasing incidence with nearly 878.348 new cases and 444.347 new death worldwide [1]. However, early diagnosis and modern therapies increase the chance of recovery [2]. On the basis of this, there must be a particular focus on alterations of the oral cavity and surrounding structures at every dental appointment.

There are various reasons that make prosthetic rehabilitation of a patient with a head and neck tumor even more difficult for the treating dentist. Restoring the aesthetics as well as the ability to swallow, chew, and speak in a satisfactory way is often a big challenge, especially after major tumor surgery [3, 4]. Depending on type, size, and location of the tumor, surgical tumor resection and subsequent reconstructive surgery followed by radiotherapy and chemotherapy often becomes necessary. This mainly results in massive changes of the anatomical conditions and also a reduced muscular activity in this area. Due to previous extensive tumor therapy, the patients are often in a poor general condition and their ability to swallow, speak, or chew is often severely hampered [5, 6]. Likewise, xerostomia is a very common negative side effect of radiotherapy for both, the patient and the treating dentist, and must be considered before prosthetic treatment [5, 7–9].

The type of dental prosthesis used depends on the oral conditions of each patient after tumor therapy (e.g., existing residual teeth extend and localization of bone and soft tissue defects, hyposalivation), compliance, and patients’ demands [3, 4]. Whenever possible, a treatment with fixed dental prosthesis should be preferred, to receive a high oral comfort. This may include the insertion of dental implants, if the existing residual teeth are not enough to attach fixed dental prosthesis. Nevertheless, there is no reason why removable dental prosthesis should not be inserted in these patients. Telescopic-retained dental prostheses show excellent results also in patients with head and neck tumors [10]. Here, too, dental implants are often helpful to attach the prostheses. If covering of the defect in the maxilla after tumor surgery is not possible for any reason, obturators become necessary [11, 12]. Dental implants are often the method of choice for prosthetic treatment after resective surgery in patients with head and neck tumors with good clinical results [3, 4, 10–29].

Since the current literature is predominately focused on the survival of dental implants in patients with head and neck tumors, there is scarce information about the performance of different types of dental prosthesis as well as complications and necessary aftercare measures.

### Objectives of the study

It is the aim of the present study to investigate the clinical performance of different types of dental prosthesis. Furthermore, necessary aftercare measures and complications should be investigated. Therefore, it should be analyzed if different patient-related factors significantly influence the clinical outcome of dental prosthesis in patients with head and neck tumors. This should help to determine the influence of various clinical factors on the survival of the dental prosthesis. Furthermore, the identification of these factors can help to develop treatment strategies and aftercare concepts in order to ensure satisfying survival times for the dental prosthesis.

## Materials and methods

### Study design and setting

Using computer-based electronic health records of the Department of Prosthodontics, Justus-Liebig University Giessen, Germany, for data collection, this retrospective cohort study observed all patients with head and neck tumors who are provided with dental prostheses at the department between January 2004 and 2020.

All patients are examined at the department before tumor therapy started. At this appointment, a complete screening of the oral cavity takes place. With regard to the following prosthetic treatment, extend of the tumor, bone quality, and potential limitations of the oral structures, as well as existing teeth and implants are assessed critically. Following the first screening, an overall treatment plan is created in close cooperation with all treating doctors (e.g., surgeons, radiotherapists, and chemotherapists). After main tumor therapy was performed, all patients are examined again at the department with regard to the prosthetic treatment planned before. If the patient underwent reconstructive surgery, attention must be paid to the strength and durability of bone and soft tissue transplants. Early loading of these transplants should be avoided to prevent any kind of failure and losses. Therefore, in these cases, prosthetic rehabilitation should begin 8 to 12 months after surgery or the last radiation took place at the earliest. All patients undergo an oral hygiene program before treatment starts. Prior to a treatment with fixed dental prosthesis (following minor tumor surgery), the condition of the abutment teeth (loosening, bone loss, decay, and vitality) and possible limitations of the oral structures are evaluated again. Here, the prosthetic treatment does not differ from the regular approach. If a treatment with removable dental prosthesis is planned (following minor or major tumor surgery and/or reconstructive surgery), any restrictions in mouth opening, mobility of tongue and lips, defect size and location, bone quality, condition of oral mucosa, and signs of xerostomia are checked. In addition, before a treatment with telescopic crown-retained dental prostheses, the condition of the abutment teeth is checked like mentioned before. Also here, the prosthetic treatment does not differ from the regular approach. After major tumor surgery and following reconstructive surgery (bone and soft tissue) frequently, a treatment with dental implants is inevitable to attach dental prosthesis. Often telescopic crown-retained dental prostheses are preferred to supply edentulous patients because they are easy to handle and clean, though, especially after major bone reconstruction, a reduction of the mouth opening (vertical height) can be expected which often results in problems with the prosthodontic treatment which also has to be considered. Obturators become necessary if covering of the bone defect in the maxilla is not possible. These can be attached using implants or resisting teeth or, if the bone is adequate and the size of the bone defect is small, a full denture can be manufacture. However, telescopic crown-retained attachments on implants and or teeth should be preferred for better stability, oral comfort, and hygiene.

Solely experienced dentists performed treatment, and all dental prostheses are manufactured at the same dental laboratory. After completion of the treatment, all patients were offered to participate in a six-monthly recall program carried out by the dentist who performed the prosthetic treatment before. After a 30-day adaptation phase, all aftercare measures and complications were taken into account. All treatment steps are documented at the exact date using the electronic patient files. The dental prostheses were indicated as in need for replacement if they were irreparably damaged or if they had to be remade due to a change in the oral situation (e.g., further surgery due to tumor recurrence or implant or tooth loss). In the case a group 4 restoration was changed into a group 1, group 2, or group 3 restoration (e.g., following later reconstructive surgery and or implantation or the patient decided for a subsequent treatment with implants), the former prosthesis was also indicated as in need for replacement.

### Participants

All patients with a previous history of head and neck tumors and who are provided with dental prosthesis at the Department of Prosthodontics, Justus-Liebig University Giessen, Germany, between January 2004 and 2020 are included in the present study. The patients with dental prosthesis are divided into four groups. Group 1 includes types of fixed dental prosthesis, e.g., crowns and bridges. Group 2 contains types of removable dental prosthesis, e.g., full dentures and telescopic crown-retained dental prostheses. Group 3 comprises all types of implant-supported dental prosthesis including fixed and removable implant-supported dental prostheses. Within group 3, three subgroups are defined for statistical analysis of the survival of dental implants: group 3a (implant-supported fixed dental prosthesis); group 3b (implant-supported removable dental prosthesis), and group 3c (implant-supported obturators). Within group 4, wrought wire clasp anchored prostheses and obturators are summarized.

All patients are offered to attend in a six-monthly check-up program. During this session, the dental prosthesis is checked regarding their functionality and accuracy of fit as well as possible damage. All fixed dental prostheses are checked with regard to occlusal interferences, loosening of the restorations, and damage of the ceramic veneering. Removable dental prosthesis are checked regarding occlusal interferences, fit of the prostheses base, any possible damage of the acrylic material or problems with the attachment systems (telescopic crowns), and possible pressure spots. The abutment teeth in booth groups are also checked regarding their vitality, loosening, and caries, and X-rays are taken if necessary. Fixed and removable implant-supported dental prosthesis is checked in the same way; additionally, the implants are examined regarding periimplantitis and loosening (X-rays are taken if necessary). An extensive examination of the oral cavity of all patients takes place at every appointment. All patients with a history of head and neck cancer follow an additional annual check-up at the Department of Oral and Maxillofacial Surgery, Justus-Liebig-University Gießen, Germany.

### Variables

The following variables are set and tested as possible influencing factors on the survival of the dental prosthesis in patients with head and neck tumors: patients’ sex (female or male); type of dental prosthesis (fixed, removable, implant-supported dental prosthesis or obturators); opposing dentition (fixed, removable, implant-supported, or natural dentition); localization of the dental prosthesis (maxilla or mandible); regular participation in follow-up program or not; and any previously performed reconstructive surgery (bone and/or tissue) or not.

### Statistical methods

A Kaplan–Meier estimate with 95% confidence intervals (Cl) was conducted to calculate the survival probabilities. A cox regression was performed as well.

Start point values and target events for calculating the survival probabilities are set as follows: incorporation date and replacement date as well as date of first aftercare measure or complication of the dental prostheses, insertion date, and date of the implant removal. If none of the target events occurred, the date of the last visit of the patient was set as target event. In the case that one patient received more than one dental prosthesis, each prosthesis was considered a separate and independent case.

Using the log-rank test (*p* < 0.05), the variables mentioned before were analyzed as covariates.

## Results

### Participants

An initial search identified 165 patients who suffered from head and neck tumors and who are subsequently provided with dental prostheses. Eighteen (%) patients had to be excluded from the study because of missing follow-up data. Therefore, a collective of 147 patients with a total of 279 dental prostheses were included in the study (90 men and 57 women, mean age 60.4 ± 12.8 years).

### Descriptive data

The mean observation time was 2.7 ± 3.0 years (maximum 14.8 years).

A total of 51.6% of all dental prostheses are located in patients with squamous cell carcinoma, 5.0% in patients with pharyngeal carcinoma, 4.3% in patients with laryngeal carcinoma, and 2.5% in patients with salivary gland tumors (Table 1).

**Table 1:** Type of head and neck tumor

Type of head and neck tumor	Number (*n*)	Percent %
Squamous cell carcinoma	144	51.6
Pharyngeal carcinoma	14	5.0
Laryngeal carcinoma	12	4.4
Salivary gland tumors	7	2.5
Ceratocystic odontogenic tumor	5	1.8
Ameloblastoma	5	1.8
Melanoma	3	1.1
Tonsil cancer	3	1.1
Others	11	
Unknown	75	26.9
Total	279	100

The types of dental prostheses are distributed as follows: 3.6% (*n* = 10) fixed dental prostheses (group 1), 26.5% (*n* = 74) removable dental prostheses (group 2), 37.6% (*n* = 105) implant supported dental prostheses (group 3), and 32.2% (*n* = 90) prostheses anchored using wrought-wire clasps or obturator prostheses (group 4). For 105 implant-supported dental prostheses, a total of 488 dental implants are set.

One hundred forty-four dental prostheses are located in the maxilla and 135 in the mandible. The opposing dentition is distributed as follows: 26.2% fixed dental prostheses or natural dentition (*n* = 73), 40.9% removable dental prostheses (*n* = 114), 23.7% implant supported dental prostheses (*n* = 66), and 9.3% wrought-wire clasp anchored prostheses (*n* = 26).

Reconstructive surgery was previously performed in 113 (40.5%) cases (Table 2).

**Table 2:** Type of reconstructive surgery

Type of reconstructive surgery	Number (*n*)	Percent %
Fibular graft	58	20.8
Radial flap surgery	25	9.0
Iliac crest augmentation	5	1.6
Others	3	1.1
Unknown	22	7.9
No reconstructive surgery	166	59.5
Total	279	100

### Main results

#### Survival of dental prostheses

**Fig. 1:**
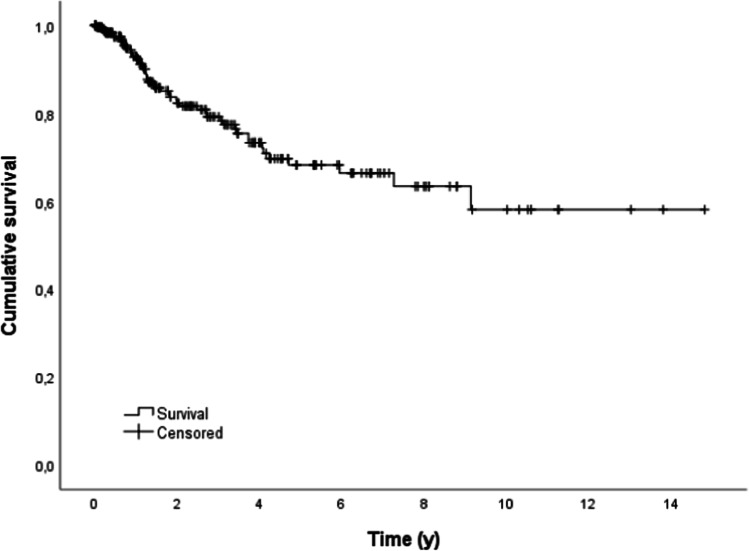
Outcome probability of all dental prosthesis (target event: replacement, *n* = 279)

A total of 49 (17.6%) dental prostheses had to be replaced. The reasons for replacement are shown in Table 3. The mean ± standard deviation (SD) expected survival time for all dental prostheses was 10 ± 0.6 years (95% CL: 8.9 to 11.3 years). After 5 and 10 years, 68.3% and 58.1% of all dental prostheses were still functional (Fig. 1).

**Table 3:** Reasons for denture replacement

Reason for denture replacement	Number (*n*)	Percentage (%)
Provisional changed into permanent denture*	32	65.3
Change in oral situation**	8	16.3
Dentures are irreparably damaged	5	10.2
Subsequently following implantation	4	8.2
Total	49	100

*After reconstructive surgery was performed and/or implants were set at a later time

**Due to tumor recurrence, tooth or implant loss

**Fig. 2:**
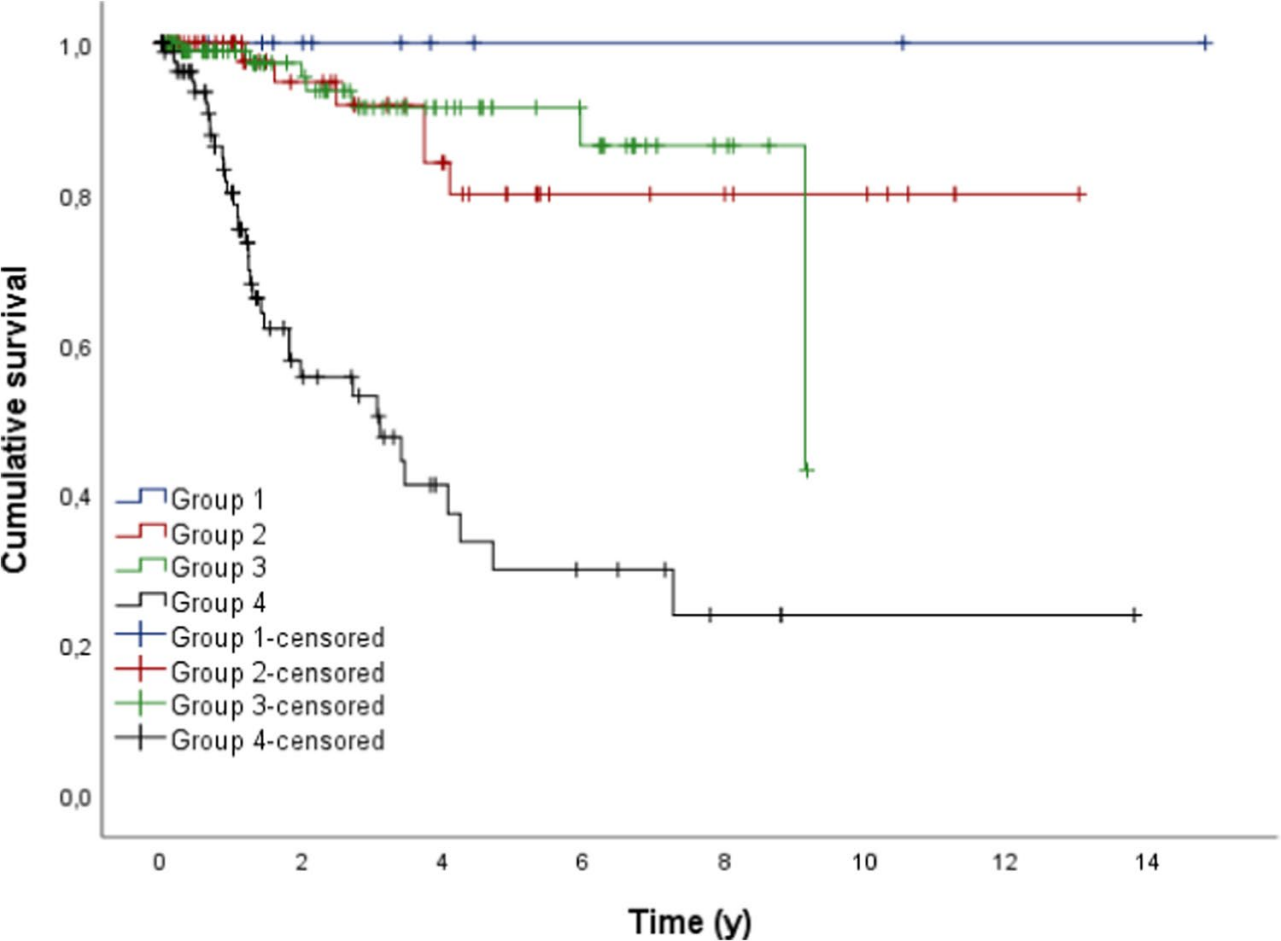
Outcome probability of all dental prosthesis dependent on the type of dental prosthesis (target event: replacement; *n* = 279)

With regard to the type of dental prosthesis, group 4 showed a significant shorter survival probability in comparison to the other groups (*p* < 0.05) (Fig. 2, Table 4). In group 1, no replacement occurred during our observation period. After 5 years, 79.9% of the dental prostheses in group 2, 91.4% of group 3, and 30% of group 4 were still functional.

**Table 4:** Mean time (y) to replacement of the different types of dental prosthesis

Type of dental prosthesis	Mean	SE	95% CL	
			Lower	Upper
Group 1*	-	-	-	-
Group 2	11.013	0.743	9.557	12.469
Group 3	8.358	0.308	7.756	8.961
Group 4	5.163	0.848	3.501	6.825
Total	9.295	0.576	8.166	10.425

^*^In group 1 no replacement occurred during our observation time

**Fig. 3:**
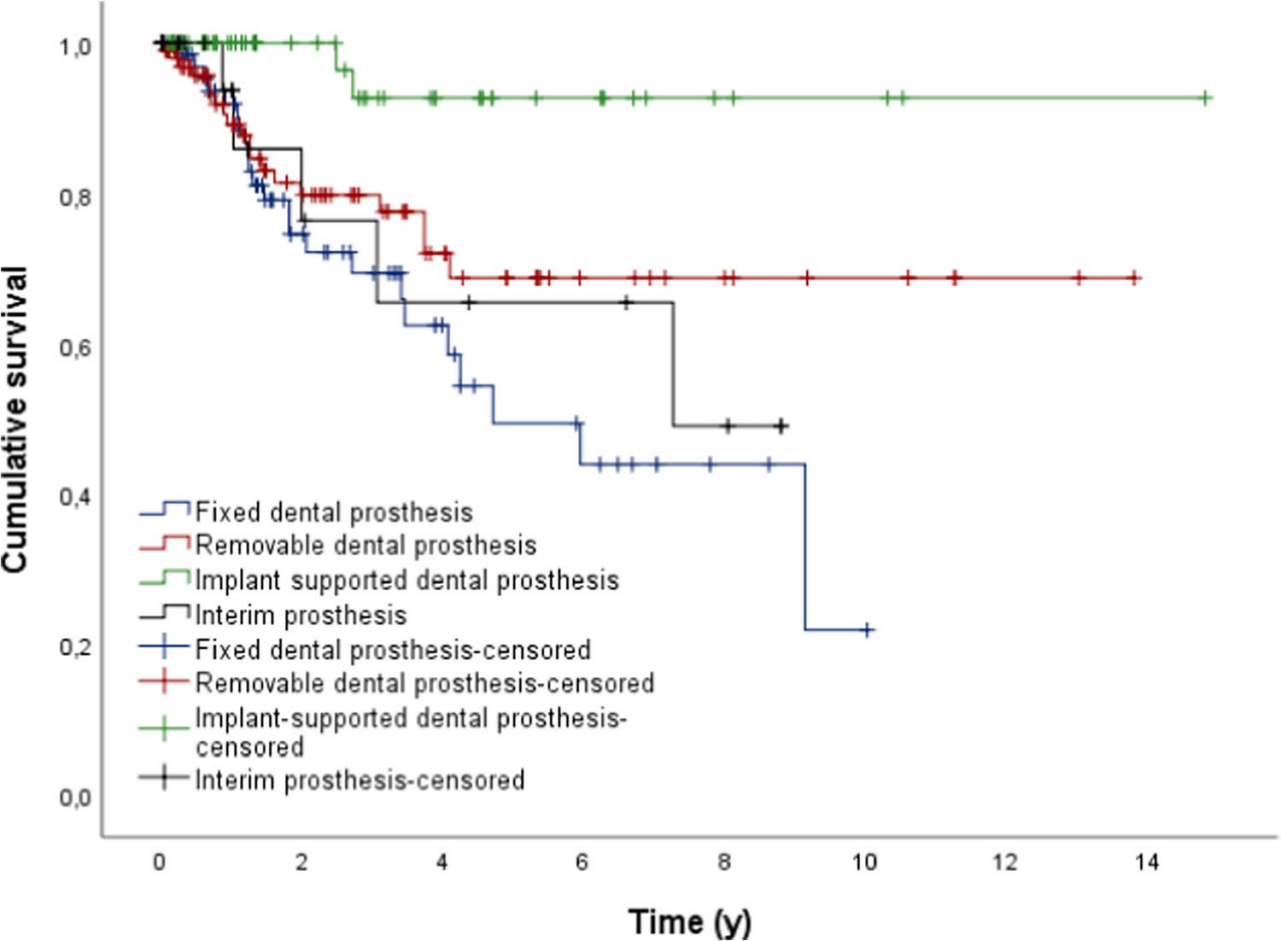
Outcome probability of all dental prosthesis dependent on the opposing dentition (target event: replacement; *n* = 279)

If implant-supported dental prostheses were located in the opposing dentition, the survival probability was significantly higher for the dental prostheses in comparison to other restorations or even no restorations (*p* < 0.05; Fig. 3, Table 5).

**Table 5:** Mean time (y) to replacement of dental prosthesis depending on the opposing dentition

Opposing dentition	Mean	SE	95% CL	
			Lower	Upper
Fixed dental prosthesis*	5.756	0.614-	4.552	6.960
Removable dental prosthesis	10.103	0.750	8.632	11.574
Implant-supported dental prosthesis	13.921	0.605	12.735	15.107
Interim prosthesis	6.167	0.957	4.292	8.043
Total	10.112	0.612	8.913	11.312

The Cox regression also showed a significant influence of the factors “type of dental prostheses” and “localization of the prostheses” on the survival probability (*p* < 0.05). Therefore, group 2 and group 3 showed a 12.5 times and 12.0 times lower risk, respectively, to fail in comparison to group 4. Dental prostheses located in the maxilla had a 57.6% lower risk to fail in comparison to prostheses located in the mandible.

None of the other factors observed showed a significant influence on the survival of the dental prostheses (*p* > 0.05).

### Survival of dental implants

Overall, 488 dental implants supported 105 (37.6%) dental prostheses. Seventy-seven (15.8%) dental implants failed during the observation period.

**Fig. 4:**
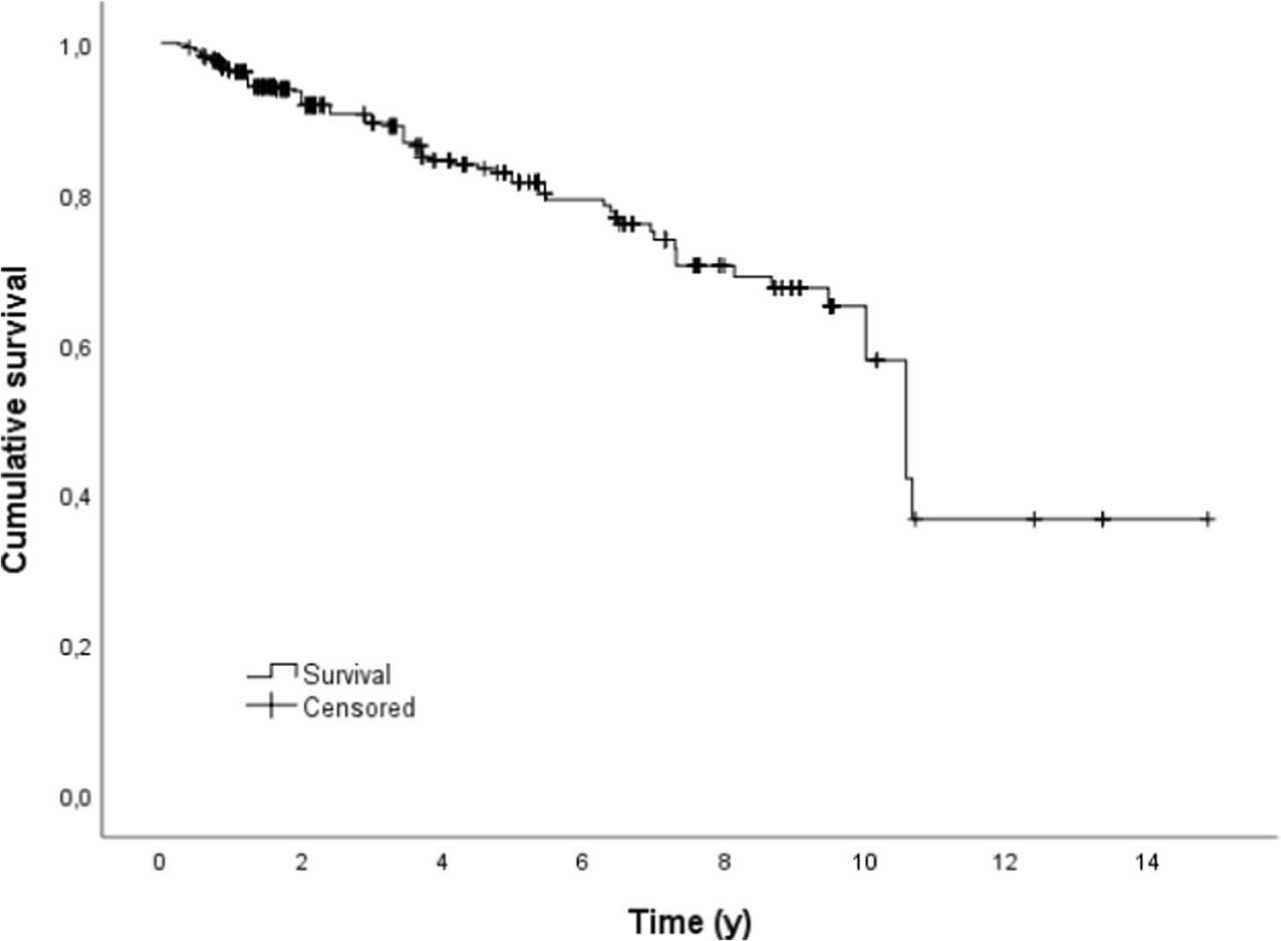
Outcome probability of all dental implants (target event: implant loss, *n* = 488)

The mean ± standard deviation (SD) expected survival time was 10.1 ± 0.5 years (95% CL: 9.1 to 11.1 years). The cumulative 5- and 10-year survival rates are 80.0% and 57.8% for dental implants, respectively (Fig. 4).

**Fig. 5:**
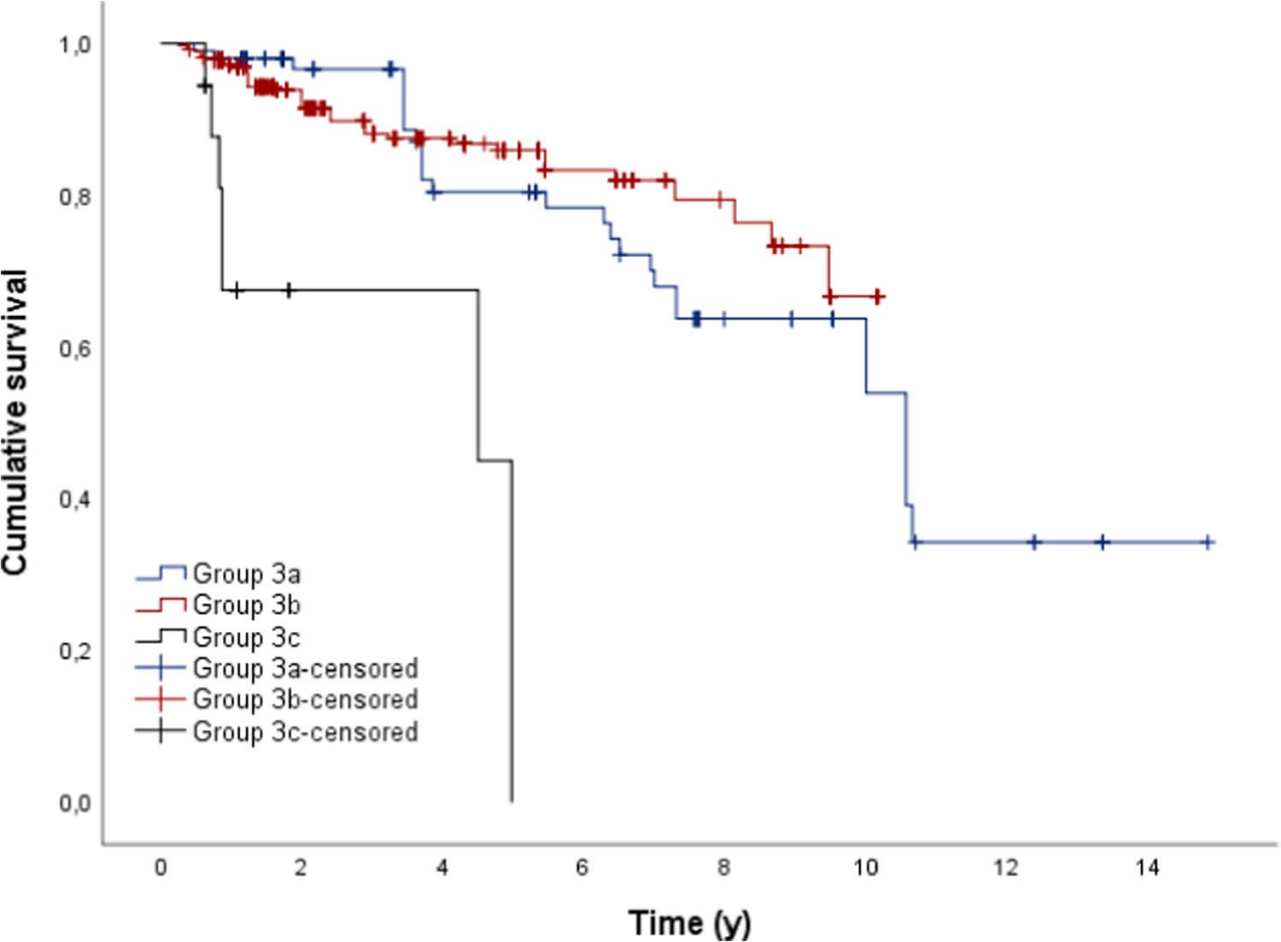
Outcome probability of all dental implants dependent on the type of restoration (target event: implant loss; *n* = 488)

Implants supporting group 3c prostheses showed significantly shorter mean survival times in comparison to the other groups (*p* < 0.05; Fig. 5, Table 6.).

**Table 6:** Mean time (y) to removal of the dental implants depending on the type of dental prosthesis

Type of implant supported dental prosthesis	Mean	SE	95% CL	
			Lower	Upper
Fixed dental prosthesis*	9.878	0.654	8.596	11.161
Removable dental prosthesis	8.611	0.224	8.172	9.050
Interim prosthesis	3.501	0.526	2.470	4.532
Total	10.091	0.496	9.118	11.064

**Fig. 6:**
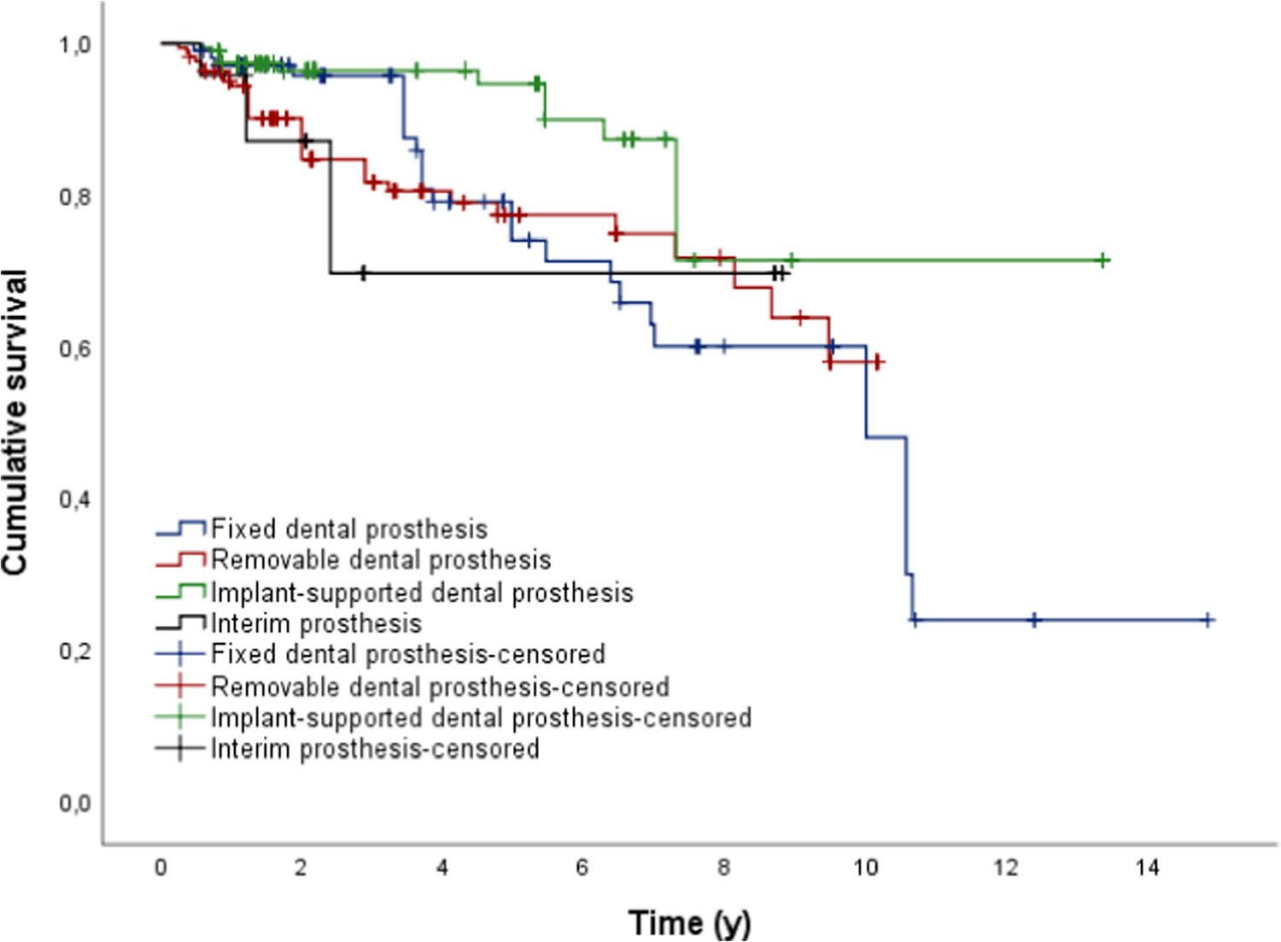
Outcome probability of all dental implants dependent on the opposing dentition (target event: implant loss; *n* = 488)

If there was an implant-supported dental prosthesis in the opposing dentition, the mean survival time of the implants observed was significantly higher than in the other groups (*p* < 0.05; Fig. 6).

Cox regression showed also a significant influence on the survival of the dental implants regarding the factors type of dental prostheses and opposing dentition (*p* > 0.05). Group 3a prostheses showed a 85.7% and group 3b a 95.7% lower risk of loss in comparison to group 3c. Dental implants that showed group 2 and group 4 prostheses in the opposite dentition showed a 5.4 times and 7.3 times higher risk to fail, respectively.

The other factors analyzed showed no significant influence on the survival of the dental implants (*p* < 0.05).

### Aftercare measures

A total of 999 aftercare measures had to be carried out on 87.1% of all dental prostheses observed. The most common aftercare measure investigated were chairside carried out denture resin repairs (45.0%), e.g., pressure spot removal or removal of sharp edges. Type and number of all aftercare measures performed can be seen in Table 7.

**Table 7:** All aftercare measures performed

Aftercare measures	Number (*n*)	Percent (%)
Denture resin repair chairside	450	45.0
Reline	164	16.4
Adjustment of occlusion and proximal contacts	110	11.0
Redesign of restoration	75	7.5
Adjustment/renewal of clasp	63	6.3
Major denture repair (labside)	45	4.5
Reattachment of screw/restoration	28	2.8
Adjustment of retention	26	2.6
Renewal of occlusal seal	24	2.4
Repair of veneering	14	1.4
Total	999	100

**Fig. 7:**
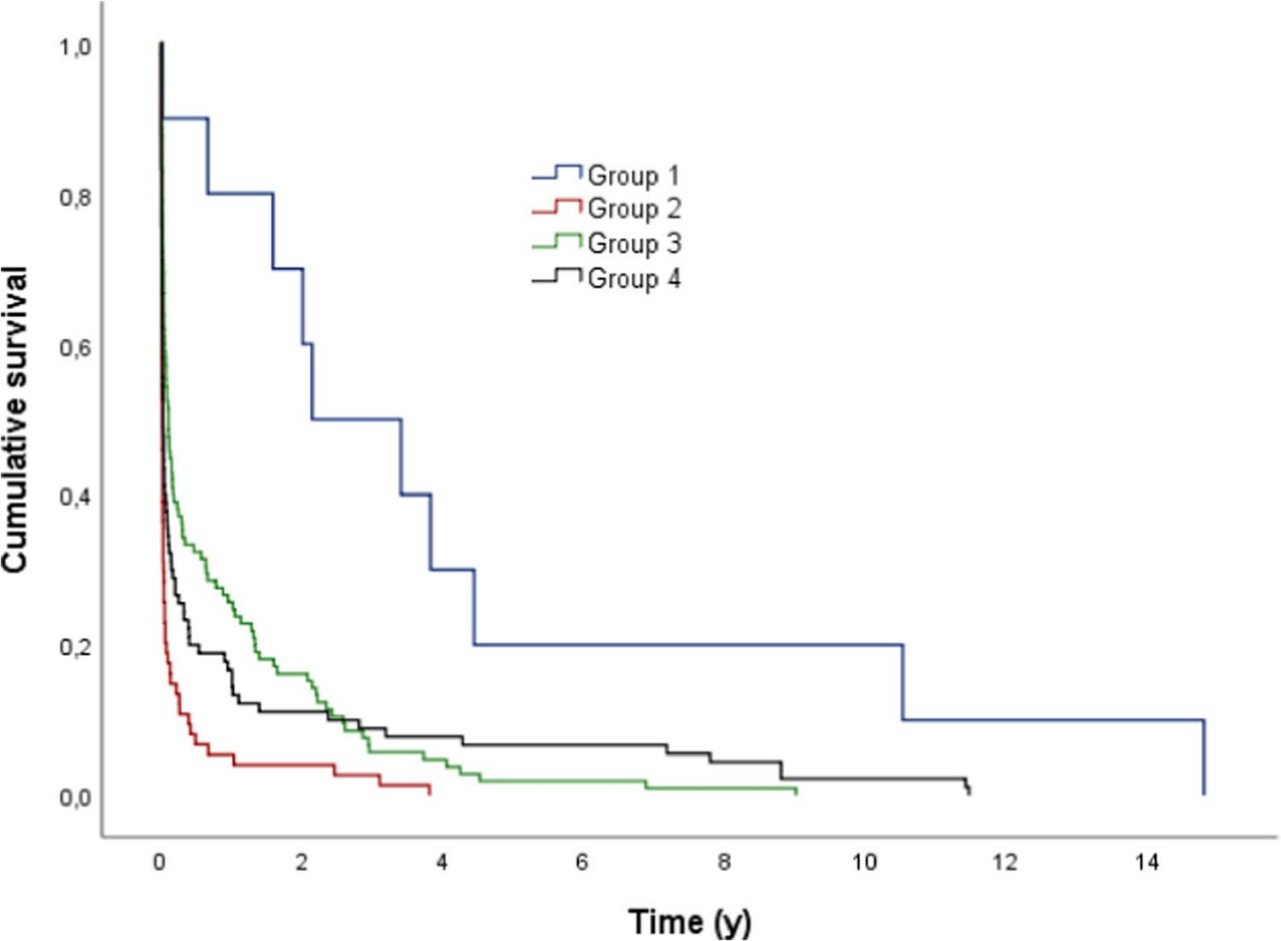
Outcome probability of all dental prosthesis dependent on the type of dental prosthesis (target event: first maintenance treatment; *n* = 999)

Group 1 showed a significantly higher survival probability until the first aftercare measurement became necessary in comparison to the other groups (*p* < 0.05; Fig. 7).

The cumulative 1-year survival time for group 1 was 80.0%, for group 2 5.4%, for group 3 25.7%, and for group 4 16.7%.

Cox regression also showed a significant influence on the time until the first aftercare measurement became necessary for the variable type of denture (*p* < 0.05). Therefore, group 1 showed a 74% lower risk regarding the time the first aftercare measure became necessary in comparison to group 4, whereas group 2 showed a 66% higher risk in comparison to group 4. Also Cox regression showed that the patient’s age significantly influenced the time until the first aftercare measure became necessary (*p* < 0.05). According to that, hazard ratio raised at 1.4% if the incorporation year of the restoration was increased by 1 year whereby the time until the first aftercare became necessary be shortened with increased age.

## Discussion

### Key results

In the current investigation, group 4 restorations showed a significantly shorter survival time after 5 years in comparison to groups 1, 2, and 3 restorations (*p* < 0.05). Therefore, after 5 years, 30% of group 4, 79.9% of group 2, 91.4% of group 3, and 100% of group 1 restorations were still functional. Cox regression also showed that the factor type of dental prostheses had a significant influence on survival (*p* < 0.05). Compared to group 4 (reference group), group 1 or group 2 restorations and group 3 restorations had a 12.5–12 time lower risk of losing their function.

Patients with implant-supported dental prostheses in the opposing dentition showed significantly higher survival times than patients with other dental prostheses or no dental prostheses in the opposing dentition (*p* < 0.05).

However, the variables patient’s sex, localization, and attendance in follow-up program as well as reconstructive surgery showed no significant influence on the survival of the dental prostheses in the present investigation.

The overall dental implant survival probability after 5 years was 80%.

Dental implants who supported group 3c prostheses showed significantly shorter survival times in comparison to implants who supported group 3a or group 3b restorations (*p* < 0.05). Dental implants in patients who also showed implant-supported dental prostheses in the opposing dentition showed significantly higher survival times in comparison to patients with other or no dental prostheses (*p* < 0.05). Here too, the other variables tested showed no significant influence on the survival of the dental implants.

A total of 999 aftercare measures had to be performed during our observation time. Group 1 restorations showed significantly higher survival times after 1 year until the first aftercare measure became necessary in comparison to the other groups (*p* < 0.05).

### Limitations

The essential aspect of the current retrospective study was to evaluate the survival of different types of dental prostheses in patients with head and neck tumors. Therefore, the 279 dental prostheses observed were divided into 4 groups. However, this presented the first limitation of the study, as it was complicated to compare the previously defined groups, and therefore also the results, with other studies. Often, only one type of dental prosthesis is considered individually in other studies [11, 12, 30, 31].

In the present study, group 4 includes prostheses anchored using wrought wire clasps as well as obturator prostheses. It should be noted that both types of prostheses are considered a temporary restoration in Germany. Therefore, it was also difficult to compare group 4 prostheses with permanent dentures (groups 1, 2, and 3) regarding their survival times as temporary restorations are usually not intended to be in the patient’s mouth for a long time. Nevertheless, they are often used as a permanent restoration in many other countries. Also in Germany, group 4 prostheses are occasionally used as permanent restorations provided that the patients can cope well with it or if a permanent restoration is not possible for various reasons at this point of treatment. It should, however, be noted that in the present study, 65.3% of all denture replacements are due to the fact that a group 4 prostheses was changed into a permanent restoration. Accordingly, there was no loss of function in these cases, but only the replacement into a permanent restoration.

In conclusion, the limitations of the current study are given by the difficulty of comparability with existing studies since these are primarily focused on survival of dental implants and mainly observed only one type of dental prostheses. Moreover, the different group sizes impeded the statistical analysis.

Of positive note is the long observation period of the current study as well as the standardized conditions of the dental prostheses regarding the manufacturing process and follow-up monitoring.

### Interpretation

A total of 17.6% of all dental prostheses observed had to be replaced in the present study. The cumulative 5-/10-year survival rate was 68.3%/58.1%. Although some comparable studies also selected the Kaplan–Meier method for analyzing survival times, these focused almost exclusively on the survival of dental implants [15, 17, 18, 32]. The survival of different types of dental prostheses in patients with head and neck tumors is almost unexplored.

During the observation period, squamous cell carcinoma was the most common malignant tumor (51.6%, see Table 1). Squamous cell carcinoma is also described in the literature as the most common tumor of the head and neck region [2].

In the present study, group 4 showed significantly shorter survival times in comparison to the other groups (*p* < 0.05). Considering the general reasons for dental prostheses replacement in the current study, the main reason for replacement was the change of a group 4 restoration into a permanent denture. The fact that group 4 prostheses were still in function after 5 (cum. survival 30%) or even 10 years (cum. survival 24%) is probably due to the fact that it was often not possible to restore these patients with permanent restorations for various reasons. Smolka et al. describe the partial lack of cooperation among patients suffering from a tumor as the main reason for the failure of the restorations [33]. The recurrence of a neoplasm in the head and neck area usually means that existing dentures have to be replaced, as a tumor resection will again result in the loss of bone and soft tissue that has to be replaced. This is also observed in other studies who also reported that a tumor recurrence is a main reason for failure [10, 33, 34]. However, this could be partially confirmed in the present study.

The reason that in group 1 no replacement occurred might be related to the fact that this group consists of 10 cases only. Moreover, patients are only provided with fixed dental prostheses if the defect of the tumor is small and the remaining teeth are in a good condition [3, 4]. It can therefore be assumed that these patients have a rather good oral hygiene, which can be a reason for the good survival times in this group too.

The cumulative survival rate after 5 years is 79.9% for group 2 prostheses. This is a rather low value in comparison to other studies, who reported survival rates of 90.0% after 5 years for removable dental prostheses [35, 36]. Nevertheless, the study of Szentpétery et al. reported similar survival times of 80.6% after 5 years for telescopic-retained dental prostheses [37]. The reason for the lower survival times in the present study can be seen in the fact that the compliance of these patients is often worse because of extensive tumor size and previous tumor therapy. Also several aggravating factors like tumor recurrences and failure of bone and soft tissue grafts can be responsible for the lower survival times [26, 33, 37]. Knowingly, there is a high risk of loss of the grafts due to a subsequent poor blood circulation [13–15, 38, 39]. Nevertheless, the variable reconstructive surgery showed no influence on the survival of the dental prostheses in the current investigation (*p* > 0.05).

Group 3 restorations showed survival rates of 91.4% and 43.2% after 5 and 10 years. In comparison, other studies reported survival times up to 100% after 5 years for implant supported dental prostheses [40–42]. However, there are also authors who reported similar survival times of 90–90.8% after 5 years [10, 43, 44]. Oral rehabilitation with implant-supported dental prostheses is not only the therapy of choice for tumor patients, but also shows good results [3, 4, 10–12, 15, 18, 25, 27, 45]. This is also confirmed on the basis of the present study.

If patients had implant-supported dental prostheses in the opposing jaw, the mean survival time of the restoration was significantly higher than with any other or no prosthetic restoration(*p* < 0.05). What was striking about this result was the fact that many patients had no restorations(*n* = 63)in the opposing jaw, but actually needed conservative and further prosthetic rehabilitation.Accordingly, a general neglect of oral hygiene can be assumed in these patients. In contrast, given the cost of implantation, it seems natural for a patient to try to maintain a high level of oral hygiene after implantation. However, comparable literature on this aspect could not be found.

There was no significant difference in the survival of the dental prostheses with regard to the factor of whether the patient received a surgical reconstruction as part of the tumor therapy or not (*p* > 0.05). This result can also confirm that a bone or tissue graft is a reliable therapeutic measure. In the literature, there are different statements about the reliability of reconstructed bone [3, 4, 12–14, 20, 22, 26, 33, 39].

The present study showed no significant difference in survival of the dental prostheses in relation to whether or not the patient participated in a regular follow-up appointment(*p* > 0.05).It should be noted, however, that participation in the recall program was very low at only 14.1%.

There was no significant difference in the survival of the dental prosthesis depending on the location of the jaw (*p* > 0.05). With regard to implant-supported dental prostheses, the literature reports a better survival of the implants and thus of the restorations located in the mandible [7, 23, 38, 46, 47]. The fact that there was no difference in the survival depending on the localization of the restorations in maxilla or mandible in the present study could therefore be due to the fact that many different types of dental prostheses were observed. Also Krennmaier et al., who observed anterior fixed partial dentures located in the mandible or maxilla, did not document any significant difference in survival [41].

The factor patient’s sex showed no influence on the survival of the restorations in the present study (*p* > 0.05), which is also confirmed by other studies [7, 10, 15, 26, 48].

Group 3 (23.7%) contained all implant-supported dental prostheses (fixed and removable), with a total of 488 dental implants. During the observation period, 77 dental implants had to be removed (15.8%). Overall, the cumulative 5-year survival rate was 80.0%, and the 10-year survival rate was 57.8% for dental implants in tumor patients. Klein et al. and Nelson et al. reported comparable 5-year survival rates of dental implants in tumor patients of 82.6% and 84%, respectively [8, 20]. In contrast, other authors observed significantly higher values with regard to the 5-year survival rate of dental implants in tumor patients, from 86.2 to 100% [3, 9, 10, 15–17, 19, 21–23, 25–29]. Furthermore, the 10-year survival rates of dental implants in tumor patients are reported from 60.3 to 98% in the literature [14, 17, 24, 26–28]. In the present study, dental implants supporting group 3c prostheses showed significantly shorter survival times in comparison to group 3a or group 3b prostheses (*p* < 0.05). This result could be due to the fact that patients who are provided with obturators often suffered from a tumor recurrence and accordingly the affected area had to be surgically resected, resulting in loss of the implant. Tumor recurrence is also reported in the literature as a common reason for failure of dental implants in tumor patients [10, 34, 49].

If patients had also group 3 prostheses in the opposing jaw, the implants showed a significantly higher survival rate (*p* < 0.05). As already mentioned, this might be explained by better oral hygiene in patients who have already been provided with implant-supported prostheses compared to patients with insufficient prosthetic restoration.

Many authors report that dental implants located in the mandible show significantly higher survival rates than in the maxilla. They argue that the quality of the alveolar bone is less favorable in the maxilla than in mandible, and bone loss has often progressed in the maxilla, so that dental implants often cannot be inserted with the correct axis [7, 9, 16, 19, 23, 38, 41, 46–48]. In the present study, the 5-year survival rate for dental implants in maxilla was 71.6% and 81.6% for mandible (*p* > 0.05). However, this result was not significant but also showed a slightly better result for implants located in the mandible.

The factor surgical reconstruction also showed no significant influence on the survival of the implants(*p* > 0.05).However, this is to be rated as positive, since implants in patients who have lost bone and have undergone reconstruction in the course of a surgery appear to have a good durability.There are studies that report a lower success rate of implants in the reconstructed bone [15, 26]. Nevertheless, in the literature, the fibular transplant as well as the Iliac crest graft show excellent results, especially in the case of large bone defects [3, 14, 38, 39, 50].

The need for aftercare measures was high in group 2. Within 1 year, 94.6% of the prostheses in this group required aftercare measures, whereas group 1 restorations showed a significantly longer survival until the first aftercare measure became necessary(*p* < 0.05). Table7shows the reasons for all aftercare measures and their percentage distribution.Forty-five percent of these aftercare measures are related to the processing and redesign of acrylic elements of the dental prosthesis (e.g., pressure spot removal), and therefore mainly refers to the group of removable dentures. However, the tumor disease also plays a decisive role in relation to the high need for aftercare of the acrylic elements of the prostheses. Regardless of whether a tumor in the jaw area occurs for the first time or it is a recurrence, the neoplasia that has occurred must be surgically removed, which, depending on the extent of the tumor, entails a redesign of a possibly existing prosthesis or possibly a completely new prosthesis.

Reline (16.4%) of the dental prostheses is the second most common aftercare measure in the present study, which is also observed in other studies [10, 51–53]. On the one hand, there is an age-related regression of the alveolar ridges over the years, which makes it necessary to reline the prosthesis; otherwise, there is a high risk of fracture for implants, teeth, and prosthesis [53]. On the other hand, especially in tumor patients, the prosthesis bases have to be adjusted accordingly after surgical procedures (e.g., tumor recurrence, tooth or implant removal).

## Conclusion

Groups 1, 2 and 3 dental prostheses in patients with head and neck tumors show slightly lower survival times than in healthy patients but they still show satisfactory results. The reasons for this can be seen in the fact that these patients often show a lower compliance and poor oral hygiene due to former tumor therapy (radiation as well as restricted mouth opening due to tumor resection). The shorter survival time of group 4 restorations is due to the fact that this type of dental prosthesis is normally not intended for permanent treatment and therefore should only be considered if a treatment with groups 1, 2, or 3 restorations is not possible for any reasons.

Dental implants in tumor patients are an adequate therapy option with good clinical results. The shorter survival times of dental implants in these patients need to be considered. Group 2 restorations showed the highest need for aftercare measures.
